# Cardinality matching versus propensity score matching for addressing cluster-level residual confounding in implantable medical device and surgical epidemiology: a parametric and plasmode simulation study

**DOI:** 10.1186/s12874-024-02406-z

**Published:** 2024-11-22

**Authors:** Mike Du, Stephen Johnston, Paul M. Coplan, Victoria Y. Strauss, Sara Khalid, Daniel Prieto-Alhambra

**Affiliations:** 1 Pharmaco- and Device Epidemiology Group, Health Data Sciences, Botnar Research Centre, NDORMS, University of Oxford, Windmill Road, Oxford, OX3 7LD UK; 2 Department of Medical Informatics, Erasmus University Medical Centre, Rotterdam, The Netherlands; 3 Epidemiology & Real-World Data Sciences, MedTech, Johnson & Johnson, New Brunswick, NJ USA; 4 Boehringer Ingelheim Pharma GmbH and Co KG, Ingelheim, Rheinland-Pfalz, DE Germany

## Abstract

**Background:**

Rapid innovation and new regulations lead to an increased need for post-marketing surveillance of implantable devices. However, complex multi-level confounding related not only to patient-level but also to surgeon or hospital covariates hampers observational studies of risks and benefits. We conducted parametric and plasmode simulations to compare the performance of cardinality matching (CM) vs propensity score matching (PSM) to reduce confounding bias in the presence of cluster-level confounding.

**Methods:**

Two Monte Carlo simulation studies were carried out: 1) Parametric simulations (1,000 iterations) with patients nested in clusters (ratio 10:1, 50:1, 100:1, 200:1, 500:1) and sample size *n* = 10,000 were conducted with patient and cluster level confounders; 2) Plasmode simulations generated from a cohort of 9981 patients admitted for pancreatectomy between 2015 to 2019 from a US hospital database. CM with 0.1 standardised mean different constraint threshold (SMD) for confounders and PSM were used to balance the confounders for within-cluster and cross-cluster matching. Treatment effects were then estimated using logistic regression as the outcome model on the obtained matched sample.

**Results:**

CM yielded higher sample retention but more bias than PSM for cross-cluster matching in most scenarios. For instance, with ratio of 100:1, sample retention and relative bias were 97.1% and 26.5% for CM, compared to 82.5% and 12.2% for PSM. The results for plasmode simulation were similar.

**Conclusions:**

CM offered better sample retention but higher bias in most scenarios compared to PSM. More research is needed to guide the use of CM particularly in constraint setting for confounders for medical device and surgical epidemiology.

**Supplementary Information:**

The online version contains supplementary material available at 10.1186/s12874-024-02406-z.

## Key messages

- Cardinality matching (CM) generally surpassed propensity score matching (PSM) in sample retention across most simulated scenarios.

- Despite CM retaining more sample, PSM gave slightly less biased treatment estimates, in most scenarios.

- Within cluster CM should not be used for data with small cluster sizes.

**Supplementary Information:**

The online version contains supplementary material available at 10.1186/s12874-024-02406-z.

## Introduction

Observational studies using routinely collected data from health and insurance registries are often used for comparative safety or effectiveness studies when randomized control trials are unfeasible or unethical [1, 2]. One key challenge for observational studies is the presence of residual confounding, arising from imbalances in patient, physician (e.g. surgeon), and hospital features [3] between treatment groups. This confounding can introduce bias in treatment effect estimation studies. Therefore, it is essential to apply statistical methods to balance the confounding covariates to avoid biased treatment estimates [4].

Propensity score matching (PSM) [5, 6] is a widely used method used in practice to balance the confounders between treatment groups in observational studies. However, PSM does not always ensure the desired balance on the original covariates or give good sample retention. These limitations can lead to bias and higher standard error for the estimation of treatment effects [7, 8]. In response, cardinality matching (CM) has been proposed as a novel method to address these issues [9]. CM uses integer programming [10, 11] to identify the largest matched sample that satisfies pre-established criteria for covariate balance. Unlike PSM, which achieves covariates balance through matching on the propensity scores, CM matches directly on the original covariates [12]. While several studies have demonstrated the advantage of CM over PSM in terms of covariate balance and sample retention [9, 13, 14], its application in observational studies with cluster-level confounding, particularly common in medical device and surgical epidemiology studies [15], has not been extensively explored. In these studies, factors such as surgeon experience and the hospital setting can influence both treatment allocation and outcomes [16].

This paper presents two different simulation studies comparing the accuracy and precision of treatment effect estimates obtained from CM and PSM. We specifically focus on two-level clustered data and binary treatment outcomes, which are typical in observational studies related to medical devices and surgical epidemiology.

## Methods

### Monte Carlo simulation data generation process

The simulation settings were based on previous simulation studies with clustered data [17, 18], but with parameters chosen to mimic the structure of a clinical dataset described below. We simulated the datasets via Monte Carlo simulations [19] with a fixed sample size of 10,000 individuals to represent the patients, binary treatment allocation (T), and binary outcome (Y). The datasets contain seven patient-level covariates (× 1 to × 7), two cluster-level covariates (z1 and z2 to represent potential hospital-level or surgeon-level confounders), and a cross-level interaction term between the individual and cluster-level confounders, which were simulated for each patient. Among the individual covariates simulated, 5 were confounders (× 1- × 5), one (× 6) was an instrumental variable associated with the treatment but not with the outcome (other than through the treatment), and × 7 was a risk factor associated with the outcome but not with treatment [20]. Both cluster-level covariates (z1 and z2) were generated as confounders, associated with treatment and outcome. The cluster and patient-level covariates were simulated from different probability distributions to reflect different covariates observed in real-world medical devices or surgical data.

**Fig. 1:**
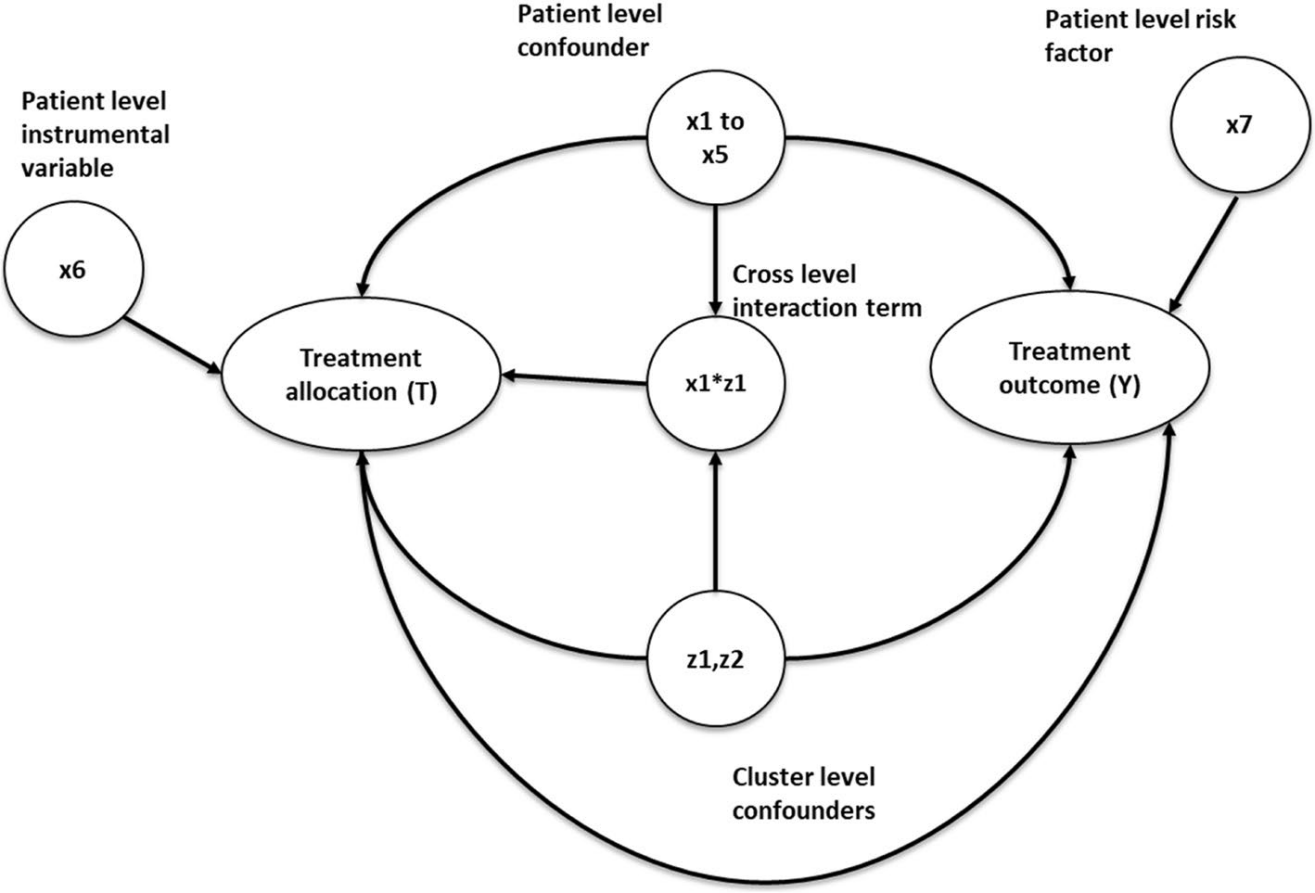
This diagram gives the causal relationship between the covariates in the simulation, the arrow indicates causes. For example, × 1- > Y implies × 1 causes Y

Twenty different scenarios were simulated to test the performance of the proposed cardinality matching balance criteria strategy. The scenarios were generated by varying the cluster structure of the data and the effect size of the cluster level confounders (z1 and z2), ranging from negligible with odds ratio = 1.01 to odds ratio = 2.5 to resemble strong cluster level confounding. Five different cluster structures were simulated with different cluster numbers (m) and average patients per cluster (n) (m = 10, *n* = 1000), (m = 50, *n* = 200), (m = 100, *n* = 100), (m = 200, *n* = 50) and (m = 500, *n* = 20). Patients per cluster (n) were randomly sampled from the Poisson distribution with mean n for each cluster within the dataset. Table 1 gives the 20 different simulation data scenarios generated. Figure 1 gives the causal diagram of the simulation covariates and the simulations are run for 1000 repetitions.

**Table 1:** The table gives the generation distribution, effects on treatment allocation and effects on treatment outcome for covariates generated in the simulations. OR = odd ratio

Covariates	Description	Effects on treatment allocation	Effects on treatment outcome	Generation distribution
**Cluster structure (m,n)**	m = number of cluster in the data n = average number of patients per a cluster	N/A	N/A	m = fixed number with 10,50,100,200,500 n = poisson(1000,200, 100, 50, 20)
**z1,z2**	Cluster-level confounders	z1 = z2 = 0.4055 (OR = 1.5)	z1 = z2 = [0.01,0.2231,0.4055,0.9163] ~ [OR = 1.01,1.25,1.5,2.5]	z1 = normal(0,1), z2 = Bernoulli(0.5)
**× 1 to × 5**	Individual level confounders	[× 1, × 2, × 3, × 4] = [0.35,0.4,0.45,0.55]	[× 1, × 2, × 3, × 4] = [0.35,0.4,0.45,0.55]	[× 1, × 2, × 3] = Bernoulli ([0.4,0.45,0.5]) × 4, × 5 = normal(0,1)
** × 6**	Individual level risk factor	0	0.5	Bernoulli( 0.5)
** × 7**	Individual level instrumental variable	0.5	0	Bernoulli( 0.5)
z1* × 1	Cross level interaction term	0.4055 (OR = 1.5)	0	z1* × 1

### Plasmode simulation data generation process

Plasmode simulation [21–23] is a method that generates synthetic data by re-sampling from pre-selected observed covariates of a real-world dataset. The re-sampling of covariates was performed using the bootstrap with replacement method [24]. The exposure and outcome of plasmode simulation data are generated using the investigators’ pre-specified re-sampled covariates from the real-world data cohort and choice of true treatment effects. Hence, simulation data generated using plasmode simulation will preserve the data structure and covariates of the real-world data cohort from which it generated the simulated data. The covariates in plasmode simulation are more closely matched to real-world data than the Monte Carlo simulation. However, plasmode simulation lacks the ability to change its data structure.

The real-world data cohort we generated in our plasmode simulation were from the US Premier Healthcare Database, an all-payer hospital database collected from among over 1000 hospitals in the US [25]. The Premier Healthcare Database includes information from hospitals’ electronic health records, including diagnoses, procedures, patient characteristics, and hospital features. The cohort included 9981 patients aged 18 or over that were admitted for pancreatectomy from 2015–2019. The patients’ covariates of the simulated data were generated based on age, sex, and Charlson comorbidity index. The cluster of the simulated data was identified using the hospital ID. The cohort had 341 unique hospital IDs, with an average of 30 patients per hospital. The cluster covariates were re-sampled from hospital-related covariates such as type (teaching or not teaching), hospital size (500 + or 500 fewer beds), and the hospital's yearly pancreatectomy volumes etc. Supplementary figure s1 and s2 provides the full list of covariates used in the plasmode simulation. The plasmode simulation is also run for 1000 repetitions.

### Propensity score matching

For propensity score matching, the propensity scores were estimated using logistic regression. Then the matched sample was selected using 1:1 ratio nearest-neighbour without replacement matching with a propensity score caliper of 0.2 standard deviations of the logit of the propensity score. This set-up for propensity score matching was chosen based on previous literature [2, 26]. Previous research has shown that a nearest-neighbour match with a caliper gives better results than other popular matching methods such as optimal matching or nearest-neighbour matching without a caliper in terms of covariate balance and bias reduction for treatment estimate [27]. The caliper width for propensity score matching was set at 0.2 standard deviations of the logit of the propensity score. This setting was used because it was shown in a simulation study by Peter Austin [2] that it tends to be optimal in terms of sample retention and bias reduction for treatment estimates. Three different propensity score matching strategies were implemented using the same matching algorithm described above. 1.) Cross-cluster matching with only the patient-level confounders included as covariates in the propensity score model. This is used as the reference and did not include any cluster-level information in the matching. 2) Cross-cluster matching, with both patient and cluster level covariates included in the propensity score model. 3) Within-cluster matching, with patient-level confounders included as covariates in the propensity score model. Since patients within the same cluster would share cluster-level covariates, including cluster-level confounders as covariates in the propensity score model for within-cluster matches is unnecessary. The selection of these matching methods was informed by Bruno Arpino et al.'s [28] simulation study, which found that within-cluster matching is optimal for larger clusters, while cross-cluster matching is preferable for datasets with smaller clusters.

### Cardinality matching

Cardinality matching was first proposed by Zubizarreta et al. [12]. It is a matching method that finds the maximum subset of patients in the treated and control groups that satisfy a set of prespecified covariates balance criteria between the treated and control group set by the investigator. The matching is done directly on the original covariates and is achieved by solving a linear integer programming problem to maximise the size of the post-matched sample. Table 2 describes the steps involved in cardinality matching.

**Table 2:** Steps involves in cardinality matching to find match sample for treatment effect estimation

*Steps involves in cardinality matching*
**1. Specify the covariate balance criteria for the post match sample. There are three elements to specify in the balance criteria** a) The covariates to balance in the post match sample between the treated and control group (e.g. all the confounders) b) A distance statistic for the balance criteria to measure the covariates balance (e.g. standardised mean difference) The maximum limit in terms of the distance statistic specified in b) for the post match sample to satisfy
**2. A matched sample with the largest possible sample size that satisfies the covariate balance criteria set in step 1 will be found using linear integer programming**
**3. The matched sample from step 2 will be rematched to minimise the covariate distances specified in step 1 between the treated and control groups.**

In this study, two cardinality matching strategies were implemented, one for within-cluster matching and one for cross-cluster matching. For cross-cluster matching, both the cluster-level and patient confounders were used as matching covariates constraints to define the balance criteria of the post-matched sample. Only the patient-level confounders were included as matching constraints for within-cluster match. To implement within-cluster match with cardinality matching, we first subset the data by cluster and then apply the same cardinality matching algorithm for each cluster to find the balanced sample. Hence the covariates selected for within cluster and across cluster match were the same between the propensity score matching and cardinality matching. All the covariates’ constraints were set using standardised mean difference (SMD) [29] with a maximum 0.1 SMD of matching covariates between the two study groups. 0.1 SMD were used as the post-match sample balance criteria because this is the standard threshold and measurement to determine whether covariates balance has been achieved for healthcare observational studies [30]. Hence, using 0.1 SMD as a matching constraint in cardinality match would always return a balanced post-match sample if it is feasible. Below is a list of all the methods compared in this study.

- *(Ref)* – Propensity score cross cluster match with patient-level confounders included as covariates in the propensity score model.
- *(PS Across)* – Propensity score cross cluster match with both patient-level and cluster-level confounders included as covariates in the propensity score model.
- *(PS Within)* – Propensity score within cluster match with patient-level confounders included as covariates in the propensity score model.
- *(CM Across)* – Cardinality matching cross cluster match with covariates constraint set on both patient-level and cluster-level confounders.
- *(CM within)* – Cardinality matching within cluster match with covariates constraint set on patient level confounders.

### Treatment effect estimation

For each of the scenarios, the average treatment effect on treated (ATT) was estimated using a logistic regression outcome model proposed by Setoguchi et al. [31]. The treatment outcome was regressed on the treatment allocation in the matched sample found with the propensity score and cardinality matching methods described above.

### Assessment of simulation results

The precision and accuracy of the estimated treatment effects for both propensity score matching and cardinality matching were compared. Using average absolute relative bias (Rbias), empirical standard error (EmpSE), 95% confidence intervals model coverage (95% Coverage) and the Monte Carlo standard error (mcse) of the 1000 repetitions for each simulated scenarios as defined in the guidance literature on simulation studies by Morris et al. [32]. Moreover, the average post-matched sample retention of the 1000 repetitions (r) for each simulated scenario was also measured and evaluated for the methods compared. The post-match sample retention is expressed as a percentage and is defined as

$$(\mathrm{Post}\;\mathrm{matched}\;\mathrm{sample}\;\mathrm{size})/(\mathrm{Maximum}\;\mathrm{possible}\;\mathrm{post}\;\mathrm{matched}\;\mathrm{sample}\;\mathrm{size})\:\times\:100$$

Since all the methods compared in this study are 1:1 without replacement matched, the maximum possible post-match sample size is two times the pre-matched sample's largest treatment group.

All analyses were performed in R version 4.3.1, with the Monte Carlo simulation data generated with the “simstudy” package [33] and the Plasmode simulation data generated with the “Plasmode” package [23]. The PSM and CM were carried out with the “Matchit” package with the “gurobi” solver [34].

## Results

**Fig. 2:**
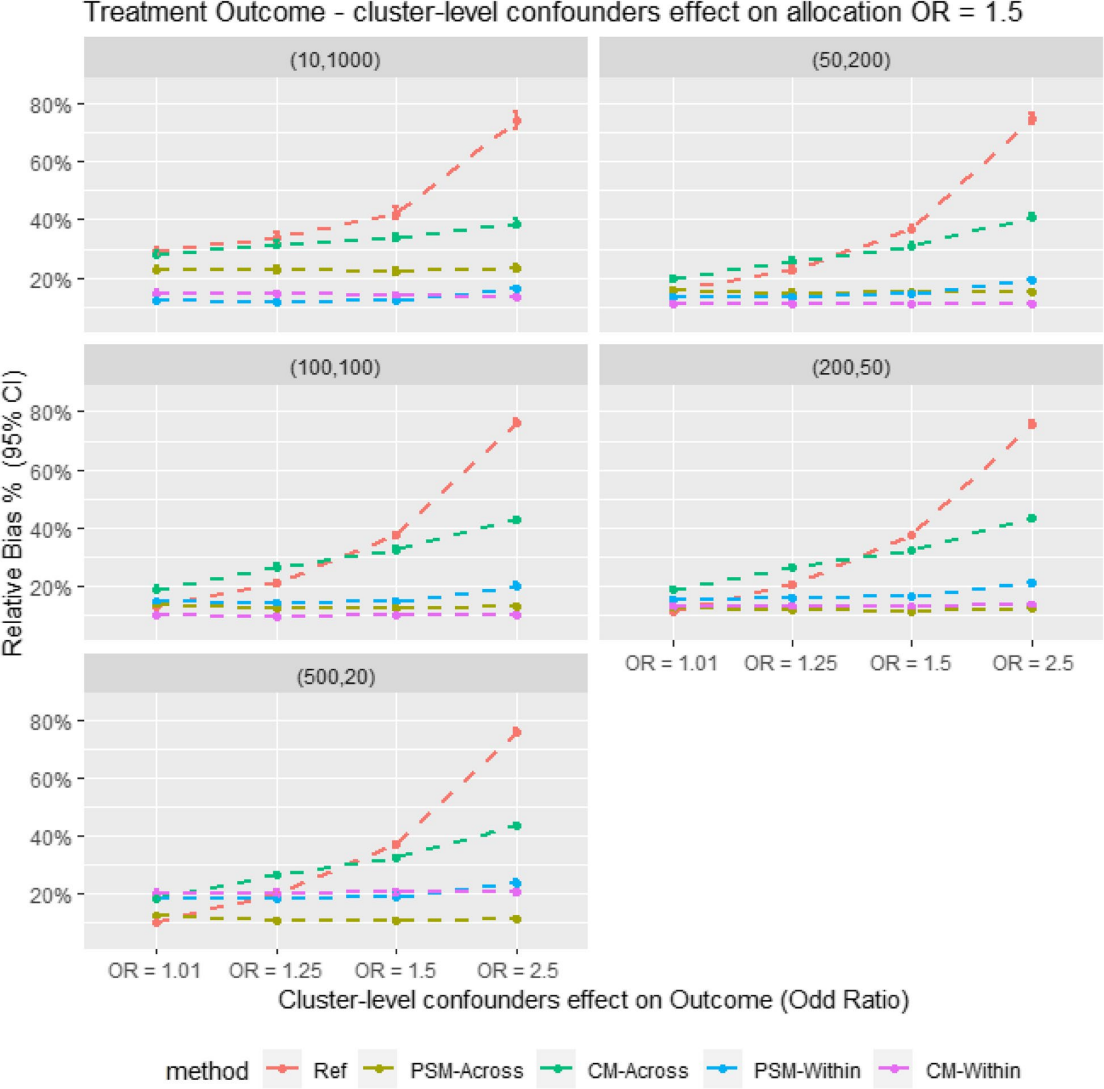
Average relative bias for different matching strategy for all the data scenario tested in the monte Carlo simulation study. E.g. Ref = Propensity score cross cluster match with patient-level confounders included as covariates in the propensity score model, PSM-Across = Propensity score cross cluster match with both patient level and cluster level confounders, PSM-Within = Propensity score within cluster match with patient-level confounders included as covariates in the propensity score model, CM-Across = Cardinality matching cross cluster match with covariates constraint set on both patient-level and cluster-level confounders, CM-Within = cardinality matching within cluster match with covariates constraint set on patient level confounders, (XX,XX) = cluster structure with ( number of cluster, average patients per cluster)

**Fig. 3:**
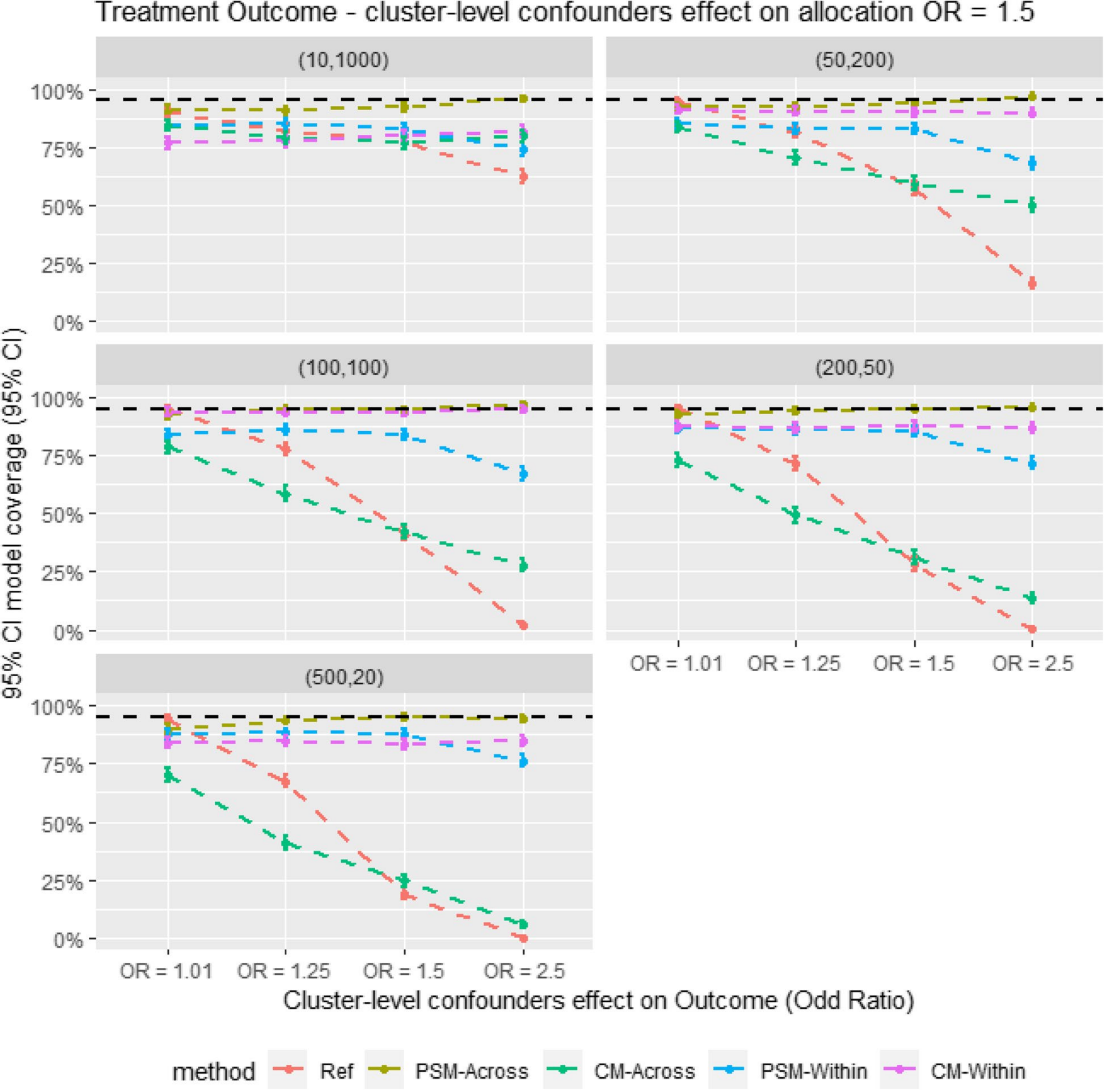
Average model coverage for different confounder effect on treatment outcome scenarios. for different matching strategy for all the data scenario tested in the Monte Carlo simulation study. E.g. Ref = Propensity score cross cluster match with patient-level confounders included as covariates in the propensity score model, PSM-Across = Propensity score cross cluster match with both patient level and cluster level confounders, PSM-Within = Propensity score within cluster match with patient-level confounders included as covariates in the propensity score model, CM-Across = Cardinality matching cross cluster match with covariates constraint set on both patient-level and cluster-level confounders, CM-Within = cardinality matching within cluster match with covariates constraint set on patient level confounders, (XX,XX) = cluster structure with ( number of cluster, average patients per cluster)

Figure 2 presents the relative bias in the Monte Carlo simulation. It shows that within-cluster matching results in lower bias compared to cross-cluster matching in scenarios with large cluster sizes. However, PS-Across gives the lowest bias in smaller cluster size scenarios (e.g., m = 500, *n* = 20). When comparing CM and PSM for within-cluster matching, CM tends to give comparable or slightly lower bias. In contrast, for cross-cluster matching, CM consistently shows higher bias than PSM across all scenarios. Figure 3 also shows a similar trend in model coverage and bias; methods with the highest bias generally have the lowest model coverage. The results are also showed in Tables 3 and 4.

**Table 3:** Result table showing average relative bias (Rbias), model coverage (Coverage) and empirical stand error (EmpSE) for different matching strategies for all the data scenarios tested in the Monte Carlo simulation study

Structure	Surgeon effect (OR)	Ref			PSM-Across			CM-Across			PSM-Within			CM-Within		
		**Rbias % (95% CI)**	**EmpSE (95% CI)**	**Coverage % (95% CI)**	**Rbias % (95% CI)**	**EmpSE (95% CI)**	**Coverage % (95% CI)**	**Rbias % (95% CI)**	**EmpSE (95% CI)**	**Coverage % (95% CI)**	**Rbias % (95% CI)**	**EmpSE (95% CI)**	**Coverage % (95% CI)**	**Rbias % (95% CI)**	**EmpSE (95% CI)**	**Coverage % (95% CI)**
**(10,1000)**	**1.01**	29.14 (27.75, 30.52)	0.148 (0.141, 0.154)	89.9 (88.03, 91.77)	22.84 (21.69, 23.99)	0.115 (0.11, 0.12)	91.1 (89.34, 92.86)	27.76 (26.53, 28.99)	0.122 (0.117, 0.127)	84.18 (81.92, 86.45)	12.2 (11.64, 12.76)	0.053 (0.051, 0.056)	84.6 (82.36, 86.84)	13.61 (13.02, 14.19)	0.051 (0.049, 0.053)	79.2 (76.68, 81.72)
	**1.25**	33.75 (32.14, 35.35)	0.157 (0.15, 0.164)	82.4 (80.04, 84.76)	22.82 (21.67, 23.96)	0.117 (0.111, 0.122)	90.7 (88.9, 92.5)	31.49 (30.15, 32.83)	0.125 (0.119, 0.13)	78.96 (76.43, 81.49)	11.77 (11.22, 12.32)	0.052 (0.05, 0.055)	84.8 (82.57, 87.03)	14.09 (13.48, 14.69)	0.051 (0.049, 0.054)	78.5 (75.95, 81.05)
	**1.50**	42.32 (40.41, 44.23)	0.164 (0.157, 0.171)	77 (74.39, 79.61)	22.27 (21.16, 23.39)	0.114 (0.109, 0.119)	92.1 (90.43, 93.77)	33.83 (32.44, 35.23)	0.123 (0.118, 0.129)	76.98 (74.37, 79.59)	12.25 (11.68, 12.82)	0.053 (0.05, 0.055)	83 (80.67, 85.33)	13.77 (13.17, 14.37)	0.051 (0.049, 0.053)	79.9 (77.42, 82.38)
	**2.50**	74.09 (71.09, 77.09)	0.217 (0.207, 0.226)	62.5 (59.5, 65.5)	23.45 (22.28, 24.62)	0.116 (0.111, 0.121)	95.8 (94.56, 97.04)	38.76 (37.22, 40.3)	0.126 (0.121, 0.132)	79.86 (77.37, 82.35)	16.35 (15.68, 17.03)	0.056 (0.053, 0.058)	74 (71.28, 76.72)	11.83 (11.27, 12.39)	0.057 (0.055, 0.06)	88 (85.99, 90.01)
**(50,200)**	**1.01**	15.71 (14.97, 16.45)	0.078 (0.075, 0.082)	94.3 (92.86, 95.74)	15.67 (14.97, 16.37)	0.072 (0.069, 0.075)	92.2 (90.54, 93.86)	19.65 (18.8, 20.5)	0.068 (0.065, 0.071)	83.7 (81.41, 85.99)	13.34 (12.73, 13.95)	0.052 (0.049, 0.054)	84.9 (82.68, 87.12)	11.01 (10.5, 11.52)	0.048 (0.046, 0.05)	90.3 (88.47, 92.13)
	**1.25**	22.71 (21.74, 23.68)	0.083 (0.079, 0.087)	81.3 (78.88, 83.72)	14.79 (14.09, 15.49)	0.071 (0.068, 0.074)	92.5 (90.87, 94.13)	25.77 (24.83, 26.7)	0.069 (0.066, 0.072)	70.6 (67.78, 73.42)	13.52 (12.9, 14.13)	0.053 (0.05, 0.055)	82.8 (80.46, 85.14)	11.6 (11.07, 12.12)	0.05 (0.048, 0.053)	89 (87.06, 90.94)
	**1.50**	36.89 (35.6, 38.18)	0.089 (0.085, 0.093)	57.2 (54.13, 60.27)	14.98 (14.28, 15.68)	0.072 (0.069, 0.076)	93.8 (92.31, 95.29)	30.86 (29.85, 31.88)	0.071 (0.068, 0.074)	59.6 (56.56, 62.64)	14.74 (14.11, 15.37)	0.053 (0.05, 0.055)	83 (80.67, 85.33)	11.19 (10.67, 11.71)	0.05 (0.048, 0.052)	89.9 (88.03, 91.77)
	**2.50**	74.75 (73.06, 76.43)	0.111 (0.106, 0.116)	16.5 (14.2, 18.8)	15.25 (14.56, 15.94)	0.069 (0.066, 0.072)	97.1 (96.06, 98.14)	41.12 (40.06, 42.18)	0.07 (0.067, 0.074)	49.8 (46.7, 52.9)	19.05 (18.33, 19.77)	0.052 (0.05, 0.054)	67.9 (65.01, 70.79)	9.14 (8.72, 9.55)	0.046 (0.044, 0.048)	95.7 (94.44, 96.96)
**(100,100)**	**1.01**	13.18 (12.58, 13.78)	0.065 (0.062, 0.067)	95 (93.65, 96.35)	13.74 (13.12, 14.36)	0.061 (0.059, 0.064)	92.3 (90.65, 93.95)	19 (18.24, 19.76)	0.059 (0.056, 0.061)	78.7 (76.16, 81.24)	14.53 (13.9, 15.15)	0.055 (0.053, 0.057)	83.8 (81.52, 86.08)	9.42 (8.99, 9.85)	0.047 (0.045, 0.049)	95.6 (94.33, 96.87)
	**1.25**	20.98 (20.12, 21.84)	0.064 (0.062, 0.067)	77.8 (75.22, 80.38)	12.2 (11.64, 12.76)	0.058 (0.056, 0.061)	95.1 (93.76, 96.44)	26.48 (25.65, 27.31)	0.056 (0.054, 0.059)	58.6 (55.55, 61.65)	14.2 (13.58, 14.83)	0.054 (0.051, 0.056)	86.3 (84.17, 88.43)	9.37 (8.92, 9.82)	0.048 (0.046, 0.05)	94.5 (93.09, 95.91)
	**1.50**	37.32 (36.3, 38.34)	0.068 (0.065, 0.071)	41.2 (38.15, 44.25)	12.1 (11.53, 12.67)	0.059 (0.056, 0.062)	94.5 (93.09, 95.91)	32.31 (31.45, 33.18)	0.058 (0.055, 0.06)	42.3 (39.24, 45.36)	14.87 (14.2, 15.53)	0.056 (0.053, 0.058)	83.9 (81.62, 86.18)	9.81 (9.34, 10.28)	0.05 (0.048, 0.052)	93.6 (92.08, 95.12)
	**2.50**	75.99 (74.76, 77.21)	0.08 (0.077, 0.084)	2.2 (1.29, 3.11)	12.9 (12.31, 13.49)	0.057 (0.055, 0.06)	96.9 (95.83, 97.97)	42.68 (41.83, 43.52)	0.055 (0.053, 0.058)	27.8 (25.02, 30.58)	20.21 (19.47, 20.95)	0.053 (0.05, 0.055)	67.2 (64.29, 70.11)	10.55 (10.06, 11.04)	0.047 (0.045, 0.05)	91.6 (89.88, 93.32)
**(200,50)**	**1.01**	11.45 (10.93, 11.97)	0.056 (0.054, 0.059)	95.2 (93.88, 96.52)	12.08 (11.51, 12.65)	0.053 (0.05, 0.055)	92.4 (90.76, 94.04)	18.71 (18.01, 19.4)	0.051 (0.049, 0.054)	72.7 (69.94, 75.46)	15.1 (14.41, 15.79)	0.058 (0.056, 0.061)	86.8 (84.7, 88.9)	12.14 (11.57, 12.72)	0.051 (0.048, 0.053)	88.2 (86.2, 90.2)
	**1.25**	20.45 (19.67, 21.22)	0.057 (0.054, 0.059)	71.6 (68.81, 74.39)	11.53 (10.99, 12.06)	0.054 (0.051, 0.056)	93.8 (92.31, 95.29)	26.41 (25.65, 27.16)	0.052 (0.05, 0.054)	49.2 (46.1, 52.3)	15.82 (15.13, 16.52)	0.059 (0.057, 0.062)	85.9 (83.74, 88.06)	12.08 (11.53, 12.63)	0.05 (0.048, 0.052)	89.7 (87.82, 91.58)
	**1.50**	37.28 (36.4, 38.16)	0.059 (0.056, 0.061)	28.1 (25.31, 30.89)	11.14 (10.62, 11.66)	0.053 (0.051, 0.055)	95.1 (93.76, 96.44)	32.27 (31.51, 33.04)	0.051 (0.048, 0.053)	31.2 (28.33, 34.07)	16.6 (15.92, 17.28)	0.059 (0.056, 0.062)	85.5 (83.32, 87.68)	12.69 (12.12, 13.25)	0.05 (0.048, 0.052)	87.6 (85.56, 89.64)
	**2.50**	75.78 (74.73, 76.83)	0.069 (0.066, 0.072)	0.30 (-0.04, 0.64)	12.19 (11.63, 12.74)	0.053 (0.05, 0.055)	95.8 (94.56, 97.04)	43.22 (42.45, 43.99)	0.05 (0.048, 0.052)	13.7 (11.57, 15.83)	21.04 (20.24, 21.84)	0.058 (0.056, 0.061)	71.7 (68.91, 74.49)	16.42 (15.77, 17.06)	0.05 (0.047, 0.052)	75.9 (73.25, 78.55)
**(500,20)**	**1.01**	10.42 (9.93, 10.91)	0.051 (0.048, 0.053)	94.1 (92.64, 95.56)	12.25 (11.7, 12.8)	0.051 (0.049, 0.053)	89.9 (88.03, 91.77)	18.14 (17.48, 18.81)	0.047 (0.045, 0.05)	70.2 (67.37, 73.03)	18.68 (17.84, 19.52)	0.074 (0.071, 0.077)	87.7 (85.66, 89.74)	19.56 (18.72, 20.4)	0.07 (0.067, 0.073)	84.6 (82.36, 86.84)
	**1.25**	20.03 (19.31, 20.74)	0.051 (0.049, 0.054)	67.6 (64.7, 70.5)	10.73 (10.24, 11.23)	0.049 (0.047, 0.051)	93.9 (92.42, 95.38)	26.54 (25.84, 27.23)	0.046 (0.044, 0.048)	41 (37.95, 44.05)	18.57 (17.76, 19.37)	0.072 (0.069, 0.075)	88.2 (86.2, 90.2)	19.7 (18.88, 20.53)	0.071 (0.068, 0.074)	84.4 (82.15, 86.65)
	**1.50**	36.93 (36.13, 37.73)	0.053 (0.05, 0.055)	19.2 (16.76, 21.64)	10.61 (10.12, 11.09)	0.049 (0.047, 0.051)	95 (93.65, 96.35)	32.37 (31.67, 33.06)	0.046 (0.044, 0.048)	24.5 (21.83, 27.17)	19.11 (18.27, 19.94)	0.074 (0.07, 0.077)	87.8 (85.77, 89.83)	20.65 (19.81, 21.5)	0.071 (0.068, 0.074)	83.2 (80.88, 85.52)
	**2.50**	75.99 (75.11, 76.88)	0.058 (0.055, 0.061)	0 (0, 0)	11.44 (10.92, 11.96)	0.048 (0.046, 0.05)	94.4 (92.97, 95.83)	43.74 (43.03, 44.44)	0.046 (0.044, 0.048)	5.5 (4.09, 6.91)	23.89 (22.95, 24.84)	0.072 (0.069, 0.075)	76.2 (73.56, 78.84)	24.77 (23.84, 25.7)	0.07 (0.067, 0.073)	73.2 (70.45, 75.95)

**Table 4:** Result table showing the average sample retention (Retention) and Monte Carlo standard error (mcse)) for different matching strategies for all the data scenarios tested in the Monte Carlo simulation study

Structure	Surgeon effect (OR)	Ref		PSM-Across		CM-Across		PSM-Within		CM-Within	
		**Retention % (95% CI)**	**mcse**	**Retention % (95% CI)**	**mcse**	**Retention % (95% CI)**	**mcse**	**Retention % (95% CI)**	**mcse**	**Retention % (95% CI)**	**mcse**
**(10,1000)**	**1.01**	88.08 (87.72, 88.44)	0.003	80.43 (79.96, 80.89)	0.003	93.73 (93.3, 94.17)	0.003	71.92 (71.45, 72.39)	0.001	80.2 (79.67, 80.72)	0.001
	**1.25**	88.14 (87.79, 88.5)	0.004	80.51 (80.05, 80.98)	0.003	93.84 (93.41, 94.27)	0.003	71.98 (71.51, 72.46)	0.001	80.2 (79.67, 80.72)	0.001
	**1.50**	88.21 (87.85, 88.57)	0.004	80.64 (80.17, 81.1)	0.003	93.87 (93.45, 94.3)	0.003	72.1 (71.63, 72.57)	0.001	80.2 (79.67, 80.72)	0.001
	**2.50**	88.18 (87.82, 88.55)	0.005	80.4 (79.92, 80.88)	0.003	93.7 (93.25, 94.14)	0.003	71.96 (71.47, 72.44)	0.001	80.2 (79.67, 80.72)	0.001
**(50,200)**	**1.01**	89.16 (89, 89.32)	0.002	82.25 (82.02, 82.48)	0.002	96.59 (96.39, 96.79)	0.002	65.62 (65.42, 65.82)	0.001	77.44 (77.19, 77.69)	0.001
	**1.25**	89.25 (89.08, 89.41)	0.002	82.41 (82.19, 82.64)	0.002	96.75 (96.55, 96.94)	0.002	65.78 (65.58, 65.98)	0.001	77.44 (77.19, 77.69)	0.001
	**1.50**	89.16 (89, 89.32)	0.002	82.23 (82, 82.45)	0.002	96.59 (96.39, 96.8)	0.002	65.6 (65.41, 65.8)	0.001	77.44 (77.19, 77.69)	0.001
	**2.50**	89.13 (88.96, 89.3)	0.002	82.24 (82.01, 82.46)	0.002	96.57 (96.36, 96.77)	0.002	65.64 (65.44, 65.84)	0.001	77.44 (77.19, 77.69)	0.001
**(100,100)**	**1.01**	89.24 (89.12, 89.37)	0.001	82.53 (82.35, 82.7)	0.001	97.05 (96.89, 97.21)	0.001	59.15 (59.01, 59.29)	0.001	74.28 (74.1, 74.45)	0.001
	**1.25**	89.29 (89.16, 89.42)	0.001	82.54 (82.37, 82.72)	0.001	97.09 (96.93, 97.25)	0.001	59.19 (59.05, 59.33)	0.001	74.28 (74.1, 74.45)	0.001
	**1.50**	89.26 (89.14, 89.39)	0.002	82.55 (82.38, 82.73)	0.001	97.09 (96.93, 97.24)	0.001	59.15 (59.01, 59.29)	0.001	74.28 (74.1, 74.45)	0.001
	**2.50**	89.35 (89.22, 89.48)	0.002	82.61 (82.44, 82.79)	0.001	97.16 (97, 97.31)	0.001	59.22 (59.09, 59.36)	0.001	74.28 (74.1, 74.45)	0.001
**(200,50)**	**1.01**	89.45 (89.35, 89.55)	0.001	82.8 (82.67, 82.94)	0.001	97.52 (97.4, 97.64)	0.001	48.99 (48.9, 49.08)	0.001	66.39 (66.27, 66.51)	0.001
	**1.25**	89.48 (89.38, 89.58)	0.001	82.83 (82.7, 82.96)	0.001	97.57 (97.46, 97.69)	0.001	49.05 (48.96, 49.14)	0.001	66.39 (66.27, 66.51)	0.001
	**1.50**	89.45 (89.35, 89.55)	0.001	82.77 (82.64, 82.9)	0.001	97.51 (97.39, 97.62)	0.001	48.99 (48.9, 49.08)	0.001	66.39 (66.27, 66.51)	0.001
	**2.50**	89.46 (89.36, 89.56)	0.002	82.81 (82.68, 82.94)	0.001	97.56 (97.44, 97.67)	0.001	49.02 (48.93, 49.11)	0.001	66.39 (66.27, 66.51)	0.001
**(500,20)**	**1.01**	89.47 (89.39, 89.56)	0.001	82.82 (82.72, 82.92)	0.001	97.63 (97.54, 97.72)	0.001	31.71 (31.65, 31.77)	0.002	33.69 (33.6, 33.78)	0.002
	**1.25**	89.46 (89.38, 89.54)	0.001	82.79 (82.69, 82.89)	0.001	97.6 (97.51, 97.69)	0.001	31.68 (31.63, 31.74)	0.002	33.69 (33.6, 33.78)	0.002
	**1.50**	89.46 (89.37, 89.54)	0.001	82.83 (82.73, 82.92)	0.001	97.62 (97.54, 97.71)	0.001	31.7 (31.64, 31.75)	0.002	33.69 (33.6, 33.78)	0.002
	**2.50**	89.45 (89.37, 89.53)	0.001	82.78 (82.68, 82.88)	0.001	97.61 (97.52, 97.69)	0.001	31.68 (31.62, 31.74)	0.002	33.69 (33.6, 33.78)	0.002

**Fig. 4:**
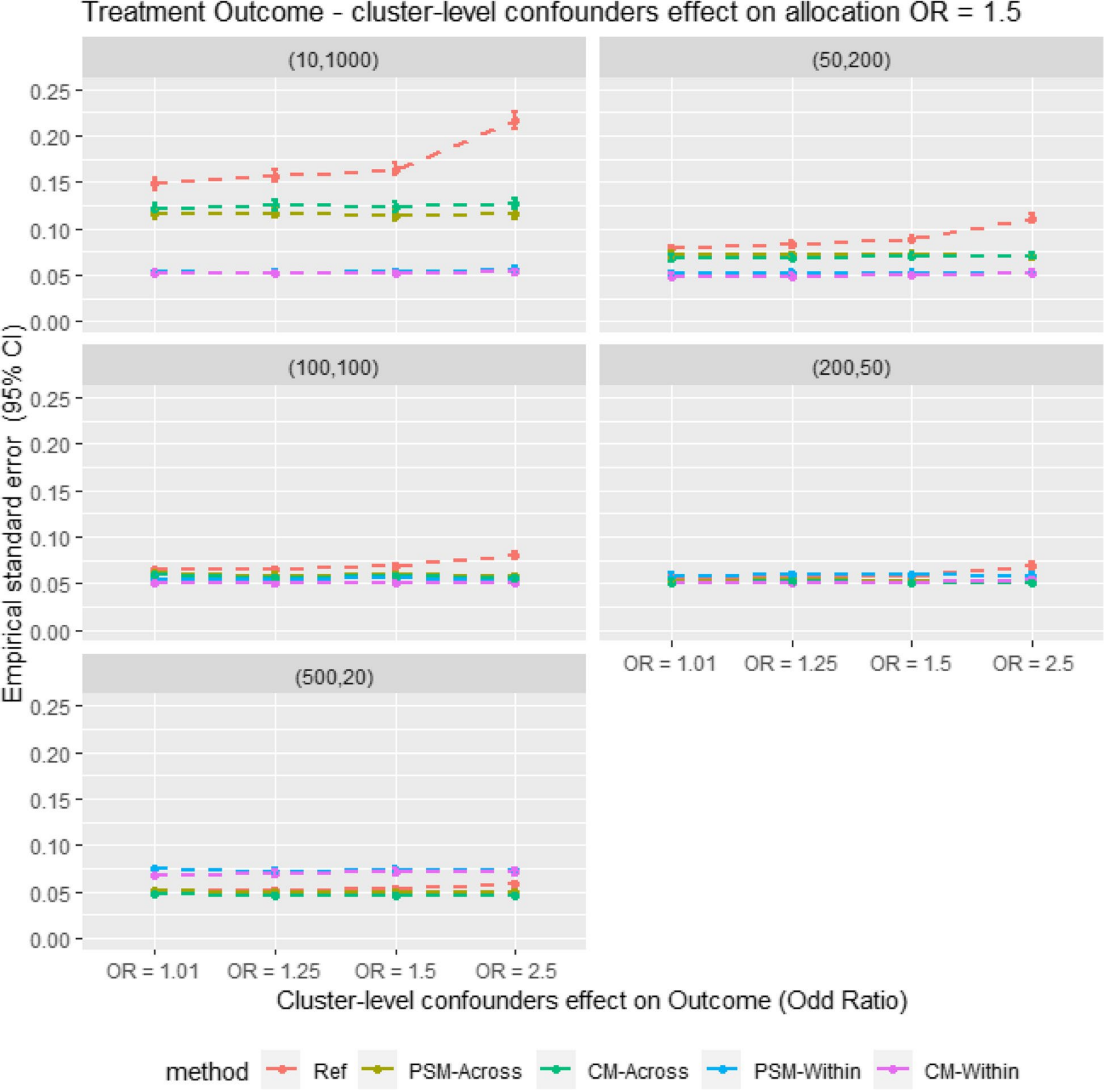
Average empirical standard error for different matching strategy for all the data scenario tested in the Monte Carlo simulation study. E.g. Ref = Propensity score cross cluster match with patient-level confounders included as covariates in the propensity score model, PSM-Across = Propensity score cross cluster match with both patient level and cluster level confounders, PSM-Within = Propensity score within cluster match with patient-level confounders included as covariates in the propensity score model, CM-Across = Cardinality matching cross cluster match with covariates constraint set on both patient-level and cluster-level confounders, CM-Within = cardinality matching within cluster match with covariates constraint set on patient level confounders, (XX,XX) = cluster structure with ( number of cluster, average patients per cluster)

As Fig. 4 shows, the empirical standard error (EmpSE) is lower for within-cluster matching than for cross-cluster matching in large cluster size scenarios (m = 10, *n* = 1000). Conversely, in smaller cluster size scenarios (m = 500, *n* = 20), EmpSE is lower for cross-cluster matching than for within-cluster matching. The EmpSE differences between CM and PSM are minimal and relatively consistent.

**Fig. 5:**
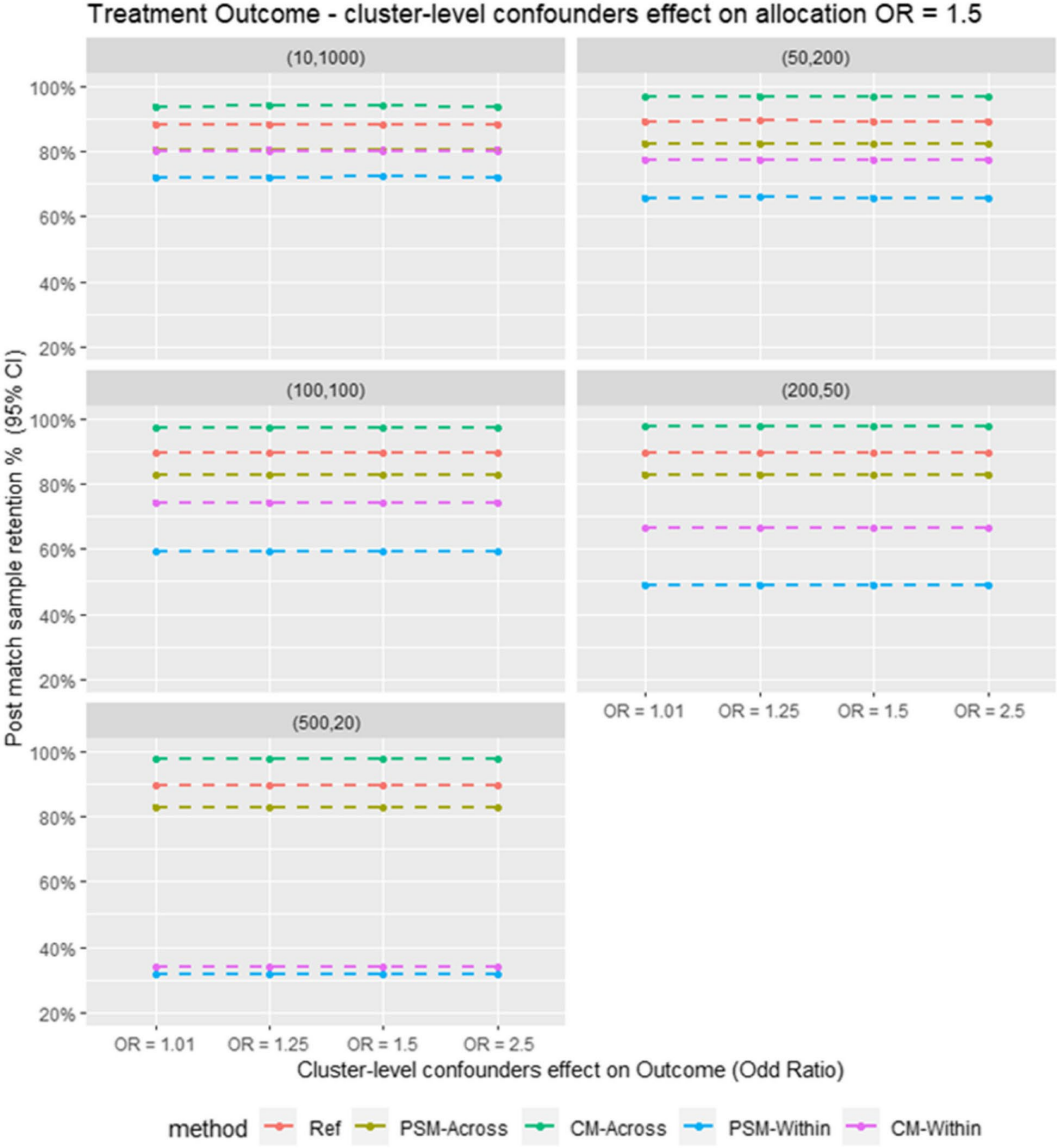
Average post match sample retention for different matching strategy for all the data scenario tested in the Monte Carlo simulation study. E.g. Ref = Propensity score cross cluster match with patient-level confounders included as covariates in the propensity score model, PSM-Across = Propensity score cross cluster match with both patient level and cluster level confounders, PSM-Within = Propensity score within cluster match with patient-level confounders included as covariates in the propensity score model, CM-Across = Cardinality matching cross cluster match with covariates constraint set on both patient-level and cluster-level confounders, CM-Within = cardinality matching within cluster match with covariates constraint set on patient level confounders, (XX,XX) = cluster structure with ( number of cluster, average patients per cluster)

Regarding sample retention as shown in Fig. 5 in the Monte Carlo simulation, CM outperforms PSM in both within-cluster and cross-cluster matching.

**Fig. 6:**
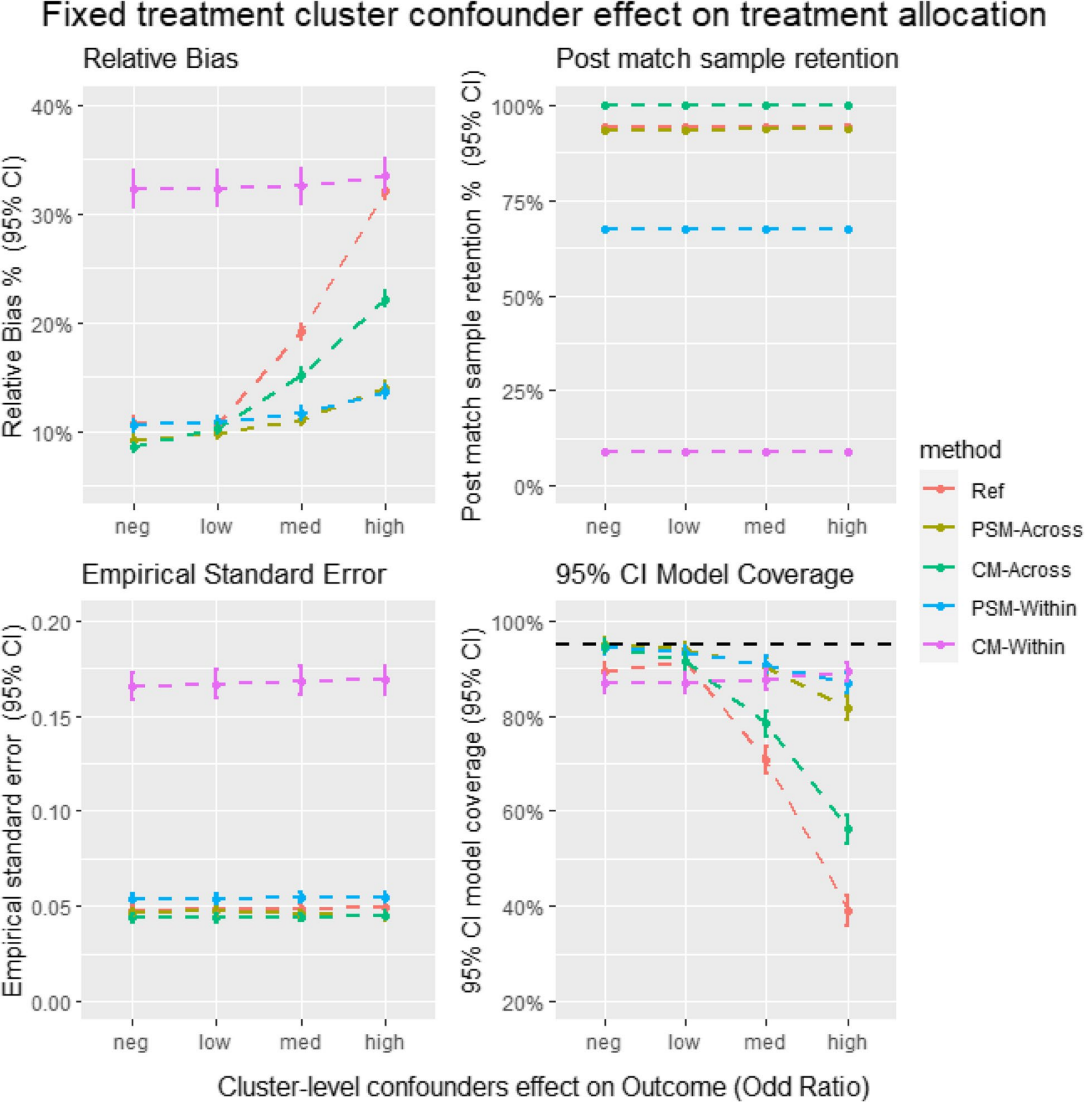
Average relative bias, model coverage, empirical stand error and sample retention for different matching strategy for all the data scenario tested in the Plasmode simulation study. Ref = Propensity score cross cluster match with patient-level confounders included as covariates in the propensity score model, PSM-Across = Propensity score cross cluster match with both patient level and cluster level confounders, PSM-Within = Propensity score within cluster match with patient-level confounders included as covariates in the propensity score model, CM-Across = Cardinality matching cross cluster match with covariates constraint set on both patient-level and cluster-level confounders, CM-Within = cardinality matching within cluster match with covariates constraint set on patient level confounders

Figure 6 provides insights into the relative bias, EmpSE, model coverage, and sample retention in the plasmode simulation. Most trends in the plasmode simulation align with those observed in the Monte Carlo simulation. For instance, the bias for CM in cross-cluster matching is higher than that for PSM, whereas CM also shows higher sample retention in cross-cluster matching. However, CM's within-cluster matching sample retention is significantly lower in the plasmode simulation compared to other methods, a trend not observed in the Monte Carlo simulation.

## Discussion

This study has several important findings regarding the performance of cardinality matching compared to propensity score matching. First, the sample retention for within-cluster match CM was higher than PSM in almost all simulated scenarios. However, PSM gave higher sample retention than CM in the plasmode simulation and similar sample retention in the smallest cluster size of the parametric simulation. A possible explanation for higher sample retention for PS-within vs CM-within in the plasmode simulation is when the match is limited to within the cluster, patient-level information is not shared across clusters in CM. In contrast, in PSM, the patient-level information was still shared across clusters through the PS model. Hence it is less likely for CM to find a feasible solution within a cluster when the cluster size is small, and as a result, all the patients within the cluster will be excluded from the post-match sample. Nevertheless, in scenarios when CM offered better sample retention than PSM when matched within the cluster, the precision and accuracy of the treatment estimate was generally better when the cluster confounding effect was strong. These results were consistent with results from previous literature, which showed that higher post-match sample retention usually gives a higher precision treatment estimate for matching analysis [14, 28].

Moreover, in cross-cluster matching CM also gave higher sample retention than PSM. However, the accuracy and precision of the treatment estimates for CM were much lower than the treatment estimates from PS. A possible explanation for a more accurate treatment estimate for PS than CM is that the balance threshold limit of 0.1 SMD might not be adequate for CM. For example, in Stephen Fortin and Stephen Johnston’s study [14], a matched sample with better covariate balance can often be found with tighter covariate constraints without much impact on sample retention. However, the current literature has limited guidelines on best practices for constraint setting for CM. Future simulation analyses testing tighter balance constraints for CM are warranted.

### Strengths and limitations

This study’s main strength is its use of simulated data, where the actual treatment effect was known. Hence using simulation studies allowed us to calculate the bias and empirical standard error for the treatment effects estimated using different matching methods. Therefore, the accuracy and precision of different methods can be compared. Using simulated data also allowed us to create different scenarios by varying data variables to see how each PS method behaves in different scenarios. This is usually difficult to achieve in real-world data analysis. Also, to our knowledge this is the first simulation study cardinality matching for a clustered observational study.

This study was also subject to limitations. A major limitation of this study is that it has not captured all cluster structure, cluster-confounding effect sizes and covariates scenarios that may occur in real-world data. Particularly, this study only tested different confounder effect sizes on treatment outcomes, and it would be interesting to consider testing different confounder effect sizes on treatment allocation in future research. Therefore, the findings may only be generalisable to the scenarios tested in this simulation study. Besides the limitation of the simulation setting, this study only tested one constraint setting for CM. It is fair to argue that better performance can be achieved by experimenting with different limits and summary measures for the constraint.

## Conclusion

This simulation study provides an insightful comparison between CM and PSM in observational studies for clustered medical device and surgical data, offering valuable perspectives on the methods' accuracy and precision across various cluster-data scenarios. The study reveals that CM maintains superior sample retention over PSM in within-cluster matching for scenarios with large cluster sizes, emphasising its effectiveness in such contexts where a robust sample size is vital for validity.

Conversely, CM's performance in small cluster size scenarios is less effective than PSM, suggesting its limited suitability for these cases. The study also highlights the necessity for further research into the optimal constraint settings for CM. This need arises from the observation that PSM consistently outperformed CM in terms of accuracy across all compared scenarios, casting doubt on the adequacy of the standard SMD = 0.1 guideline for CM.

In conclusion, the study underscores the importance of additional research to refine CM's application in the field of medical device and surgical epidemiology, prior to its broader implementation.

## Supplementary Information

Supplementary Material 1.

## Data Availability

No datasets were generated or analysed during the current study.
